# 3′-[Hy­droxy(4-oxo-4*H*-chromen-3-yl)meth­yl]-2-oxospiro­[indoline-3,2′-pyrrolidine]-3′-carbonitrile

**DOI:** 10.1107/S1600536811053098

**Published:** 2011-12-17

**Authors:** E. Govindan, K. SakthiMurugesan, A. SubbiahPandi, P. Yuvaraj, Boreddy S. R. Reddy

**Affiliations:** a Department of Physics, Presidency College (Autonomous), Chennai 600 005, India; b Industrial Chemistry Laboratory, Central Leather Research Institute, Adyar, Chennai 600 020, India

## Abstract

In the title compound, C_23_H_19_N_3_O_4_, the pyran ring adopts a half-chair conformation, while the pyrrolidine (with a C atom as the flap atom) and the five-membered ring in the indoline (with a C atom as the flap atom) ring system adopt slight envelope conformations. The pyrrolidine ring makes dihedral angles of 83.3 (1) and 60.4 (1)° with the mean plane through all non-H atoms of the indoline and chromene ring systems, respectively. In the crystal, mol­ecules are connected by two unique N—H⋯O and O—H⋯O hydrogen-bonding inter­actions, which form centrosymmetric patterns described by graph-set motifs *R*_2_^2^(18) and *R*_2_^2^(14). These two motifs combine to form a hydrogen-bonded chain which propagates in the *a*-axis direction. The crystal structure is also stablized by C—H⋯O inter­actions and by aromatic π–π stacking inter­actions between the pyran and benzene rings of neighbouring mol­ecules [centroid–centroid distance = 3.755 (1) Å and slippage = 1.371 (2) Å].

## Related literature

For general background to the biological use of pyrrolidine derivatives, see: Pettersson *et al.* (2011 ▶); Bello *et al.* (2010 ▶). For ring puckering parameters, see: Cremer & Pople (1975 ▶) and for asymmetry parameters, see: Nardelli (1983 ▶). For the structure of another pyrrolidine derivatie, see: Selvanayagam *et al.* (2011 ▶).
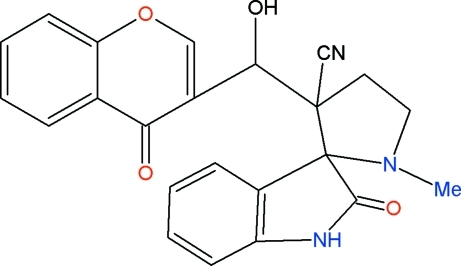


## Experimental

### 

#### Crystal data

C_23_H_19_N_3_O_4_
*M* *_r_* = 401.41
Triclinic, 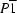

*a* = 9.3483 (7) Å
*b* = 10.2256 (9) Å
*c* = 10.9080 (9) Å
α = 71.832 (5)°
β = 88.309 (5)°
γ = 78.248 (5)°
*V* = 969.32 (14) Å^3^
*Z* = 2
Mo *K*α radiation
μ = 0.10 mm^−1^
*T* = 293 K
0.20 × 0.20 × 0.19 mm

#### Data collection

Bruker APEXII CCD area detector diffractometer
Absorption correction: multi-scan (*SADABS*; Sheldrick, 1996) ▶ *T*_min_ = 0.981, *T*_max_ = 0.982
17643 measured reflections
4841 independent reflections
3374 reflections with *I* > 2σ(*I*)
*R*_int_ = 0.028

#### Refinement

*R*[*F* ^2^ > 2σ(*F* ^2^)] = 0.039
*wR*(*F* ^2^) = 0.106
*S* = 1.02
4841 reflections
273 parameters
H-atom parameters constrained
Δρ_max_ = 0.22 e Å^−3^
Δρ_min_ = −0.21 e Å^−3^

### 

Data collection: *APEX2* (Bruker, 2007 ▶); cell refinement: *SAINT* (Bruker, 2007 ▶); data reduction: *SAINT*; program(s) used to solve structure: *SHELXS97* (Sheldrick, 2008 ▶); program(s) used to refine structure: *SHELXL97* (Sheldrick, 2008 ▶); molecular graphics: *ORTEP-3* (Farrugia, 1997 ▶); software used to prepare material for publication: *SHELXL97* and *PLATON* (Spek, 2009 ▶).

## Supplementary Material

Crystal structure: contains datablock(s) global, I. DOI: 10.1107/S1600536811053098/nk2121sup1.cif

Structure factors: contains datablock(s) I. DOI: 10.1107/S1600536811053098/nk2121Isup2.hkl

Supplementary material file. DOI: 10.1107/S1600536811053098/nk2121Isup3.cml

Additional supplementary materials:  crystallographic information; 3D view; checkCIF report

## Figures and Tables

**Table 1:** Hydrogen-bond geometry (Å, °)

*D*—H⋯*A*	*D*—H	H⋯*A*	*D*⋯*A*	*D*—H⋯*A*
N2—H2*A*⋯O2^i^	0.86	2.01	2.8479 (14)	164
O3—H3*A*⋯O4^ii^	0.82	1.97	2.7631 (14)	164
C23—H23⋯O3^iii^	0.93	2.58	3.2761 (18)	133

Symmetry codes: (i) 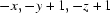; (ii) 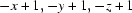; (iii) 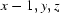.

**Fig. 1.**
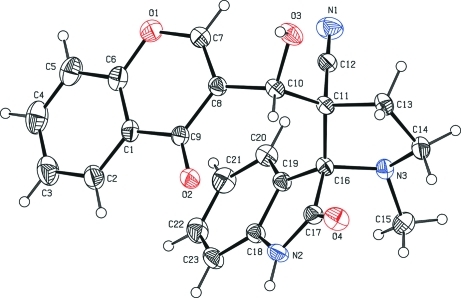
The molecular structure of the title compound with displacement ellipsoids drawn at the 30% probability level.

**Fig. 2.**
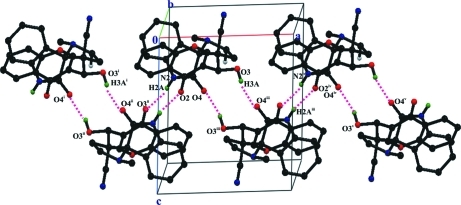
Part of the crystal packing of (I) showing N—H···O and O—H···O intermolecular hydrogen bonds showing R22(18) and R22(14) centrosymmetric dimers, respectively. [Symmetry codes: (i) -1 + x, y, z; (ii) -x, 1 - y, 1 - z; (iii) -1 + x, 1 - y, 1 - z; (iv) 1 + x, y, z; (v) 2 - x, 1 - y, 1 - z].

**Fig. 3.**
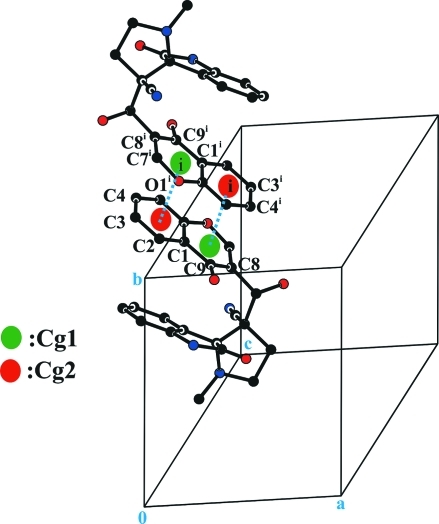
The π–π interactions shown as dotted lines in the title compound. Cg1 and Cg2 are the centroids of pyran (O1/C1/C6–C9) and benzene (C1–C6) ring, respectively. [Symmetry code: (i) -x, 2 - y, 1 - z]

## supplementary crystallographic information

### Comment

Pyrrolidine derivatives are used as norepinephrine reuptake inhibitors and 5-HT(1 A) partial agonists for treating neuropsychiatric disorders including depression and anxiety (Pettersson *et al.*, 2011). These derivatives are used as alpha-mannosidase inhibitors and with antitumor activities against hematological and solid malignancies (Bello *et al.*, 2010). In view of these importance, we have undertaken the crystal structure determination of the title compound, a pyrrolidine derivative,and the results are presented here.

Single crystal X-ray analysis confirms the molecular structure and atom connectivity as illustrated in Fig. 1. The bond lengths and angles in (Fig. 1) agree with those observed in other pyrrolidine derivatives (Selvanayagam *et al.*, 2011). The sum of the angles at N1 of the pyrrolidine ring [337.3 (2)°] is in accordance with *sp*^3^ hybridization. The pyran ring (O1/C1/C6–C9) adopts a half chair conformation with a local, non-crystallographic two fold rotation axis passing through the mid point of the [O1–C6] and [C9–C8] bonds; the puckering parameters Q, θ, φ (Cremer & Pople, 1975) and asymmetry parameter ΔC_2_[O1–C6](Nardelli, 1983) are 0.1074 (15) Å, 78.8 (8)°, 192.7 (9)° and 4.6 (2) Å, respectively. The pyrrolidine (N3/C11/C13/C14/C16) and five membered in the indoline (N2/C16—C19) rings adopt an envelope conformation with the C16 (displacement 5.8 (1) Å) and C17 (displacement 0.7 (2) Å) atoms as the flap atoms and with puckering parameters, q_2_ = 0.0888 (15) Å; φ_2_ = 326.7 (2)°; and q_2_ = 0.4459 (15) Å; φ_2_ = 38.6 (9)° respectively. The pyrrolidine ring makes dihedral angles of 83.3 (1)° and 60.4 (1)° with mean plane fitted through all non-H atoms of the indoline (N2/C16–C23) ring system and the chromen (O1/C1–C9) ring system, respectively.

In the crystal, unique N2–H2A···O2 (at *x,y,z* and *-x,1 - y,1 - z*) and O3–H3A···O4 (at *x,y,z* and *1 - x,1 - y,1 - z*) hydrogen bonding interactions form a cyclic centrosymmetric patterns, with the motif *R^2^_2_*(18) and *R^2^_2_*(14).  These combine to form a zigzag chains which propagates in the *a* axis direction (Table 1 and Fig. 2). The crystal packing is further stabilized by π–π stacking interactions between the rings *Cg*1 and *Cg*2 (at *x,y,z* and *-x, 2 - y, 1 - z*) with the centroid-centroid distances equal to 3.755 (1) Å and slippage = 1.371 (2) Å (Fig. 2; *Cg*1 and *Cg*2 are the centroids of pyran (O1/C1/C6–C9) and benzene (C1–C6) ring, respectively).

### Experimental

2-(Hydroxyl(4-oxo-4*H*-chromen-3-yl)methyl)acrylonitrile was synthesized by the Baylis-Hillman reaction of chromene-3-aldehyde, acrylonitrile and 0.1 equivalent of DABCO as a catalyst, in the presence of 1-methyl-2-pyrrolidinone (NMP) as a solvent. Baylis-Hillman adduct underwent smooth reaction with non-stabilized azomethine ylide generated from isatin and sarcosine by refluxing in acetonitrile. After that, a mixture of 2-(Hydroxyl(4-oxo-4*H*-chromen-3-yl)methyl)acrylonitrile (100 mg, 0.404 mmol), sarcosine(1.2eq), and isatin (1.2eq.) in acetonitrile(2 ml) was refluxed for 6–12 h. Completion of reaction was indicated by TLC, the solvent was then removed *in vacuo* and the crude product subjected to column chromatography (100–200 mesh) using hexane-ethyl acetate as eluent. Single crystal suitable for X-ray diffraction were obtained by slow evaporation of a solution of the title compound in hexane at room temperature.

### Refinement

H atoms were fixed geometrically (C—H = 0.93–0.98 Å, N—H = 0.86 Å and O—H = 0.82 Å) and allowed to ride on their parent atoms, with *U*_iso_(H) = 1.2*U*_eq_(C) or 1.5*U*_eq_(C, O) for the methyl and OH groups.

### Crystal data

**Table:**

C_23_H_19_N_3_O_4_	*Z* = 2
*M**_r_* = 401.41	*F*(000) = 420
Triclinic, *P*1	*D*_x_ = 1.375 Mg m^−^^3^
Hall symbol: -P 1	Mo *K*α radiation, λ = 0.71073 Å
*a* = 9.3483 (7) Å	Cell parameters from 4841 reflections
*b* = 10.2256 (9) Å	θ = 2.0–28.4°
*c* = 10.9080 (9) Å	µ = 0.10 mm^−^^1^
α = 71.832 (5)°	*T* = 293 K
β = 88.309 (5)°	Block, white
γ = 78.248 (5)°	0.20 × 0.20 × 0.19 mm
*V* = 969.32 (14) Å^3^	

### Data collection

**Table:**

Bruker APEXII CCD area detector diffractometer	4841 independent reflections
Radiation source: fine-focus sealed tube	3374 reflections with *I* > 2σ(*I*)
graphite	*R*_int_ = 0.028
ω and φ scans	θ_max_ = 28.4°, θ_min_ = 2.0°
Absorption correction: multi-scan (*SADABS*; Sheldrick, 1996)	*h* = −12→12
*T*_min_ = 0.981, *T*_max_ = 0.982	*k* = −13→13
17643 measured reflections	*l* = −14→14

### Refinement

**Table:**

Refinement on *F*^2^	Primary atom site location: structure-invariant direct methods
Least-squares matrix: full	Secondary atom site location: difference Fourier map
*R*[*F*^2^ > 2σ(*F*^2^)] = 0.039	Hydrogen site location: inferred from neighbouring sites
*wR*(*F*^2^) = 0.106	H-atom parameters constrained
*S* = 1.02	*w* = 1/[σ^2^(*F*_o_^2^) + (0.0425*P*)^2^ + 0.2158*P*] where *P* = (*F*_o_^2^ + 2*F*_c_^2^)/3
4841 reflections	(Δ/σ)_max_ < 0.001
273 parameters	Δρ_max_ = 0.22 e Å^−^^3^
0 restraints	Δρ_min_ = −0.21 e Å^−^^3^

### Special details

**Table:**

Geometry. All e.s.d.'s (except the e.s.d. in the dihedral angle between two l.s. planes) are estimated using the full covariance matrix. The cell e.s.d.'s are taken into account individually in the estimation of e.s.d.'s in distances, angles and torsion angles; correlations between e.s.d.'s in cell parameters are only used when they are defined by crystal symmetry. An approximate (isotropic) treatment of cell e.s.d.'s is used for estimating e.s.d.'s involving l.s. planes.
Refinement. Refinement of *F*^2^ against ALL reflections. The weighted *R*-factor *wR* and goodness of fit *S* are based on *F*^2^, conventional *R*-factors *R* are based on *F*, with *F* set to zero for negative *F*^2^. The threshold expression of *F*^2^ > σ(*F*^2^) is used only for calculating *R*-factors(gt) *etc*. and is not relevant to the choice of reflections for refinement. *R*-factors based on *F*^2^ are statistically about twice as large as those based on *F*, and *R*- factors based on ALL data will be even larger.

### Fractional atomic coordinates and isotropic or equivalent isotropic displacement parameters (Å^2^)

**Table:**

	*x*	*y*	*z*	*U*_iso_*/*U*_eq_	
C1	0.01655 (15)	0.90996 (14)	0.37494 (13)	0.0379 (3)	
C2	−0.12747 (17)	0.90527 (17)	0.41086 (16)	0.0507 (4)	
H2	−0.1515	0.8200	0.4571	0.061*	
C3	−0.2338 (2)	1.0263 (2)	0.37808 (19)	0.0652 (5)	
H3	−0.3298	1.0227	0.4010	0.078*	
C4	−0.1974 (2)	1.1539 (2)	0.3107 (2)	0.0701 (5)	
H4	−0.2698	1.2354	0.2897	0.084*	
C5	−0.0581 (2)	1.16225 (17)	0.27475 (17)	0.0610 (5)	
H5	−0.0347	1.2483	0.2300	0.073*	
C6	0.04833 (18)	1.03947 (15)	0.30641 (14)	0.0449 (3)	
C7	0.28974 (17)	0.93517 (15)	0.28421 (14)	0.0448 (3)	
H7	0.3811	0.9453	0.2511	0.054*	
C8	0.27088 (15)	0.80437 (14)	0.34502 (12)	0.0345 (3)	
C9	0.13386 (15)	0.78375 (14)	0.40633 (12)	0.0345 (3)	
C10	0.39326 (14)	0.67953 (14)	0.35242 (12)	0.0351 (3)	
H10	0.3858	0.6033	0.4319	0.042*	
C11	0.39211 (14)	0.62424 (13)	0.23496 (12)	0.0324 (3)	
C12	0.40376 (15)	0.74112 (15)	0.11646 (13)	0.0377 (3)	
C13	0.52387 (15)	0.50169 (15)	0.24145 (14)	0.0411 (3)	
H13A	0.6075	0.5376	0.2003	0.049*	
H13B	0.5515	0.4463	0.3304	0.049*	
C14	0.47078 (16)	0.41335 (15)	0.16957 (15)	0.0442 (3)	
H14A	0.5333	0.4056	0.0986	0.053*	
H14B	0.4697	0.3197	0.2272	0.053*	
C15	0.23476 (19)	0.40250 (18)	0.08659 (16)	0.0541 (4)	
H15A	0.2815	0.3681	0.0199	0.081*	
H15B	0.1394	0.4580	0.0558	0.081*	
H15C	0.2256	0.3245	0.1610	0.081*	
C16	0.26018 (14)	0.56017 (13)	0.21551 (11)	0.0318 (3)	
C17	0.22340 (15)	0.45055 (14)	0.34238 (12)	0.0361 (3)	
C18	0.00806 (15)	0.59547 (14)	0.25514 (13)	0.0363 (3)	
C19	0.11179 (14)	0.65327 (14)	0.17285 (12)	0.0335 (3)	
C20	0.06507 (16)	0.77525 (15)	0.07153 (13)	0.0423 (3)	
H20	0.1312	0.8141	0.0131	0.051*	
C21	−0.08275 (18)	0.83888 (17)	0.05857 (16)	0.0515 (4)	
H21	−0.1149	0.9213	−0.0088	0.062*	
C22	−0.18214 (17)	0.78190 (18)	0.14373 (17)	0.0528 (4)	
H22	−0.2799	0.8274	0.1341	0.063*	
C23	−0.13801 (16)	0.65743 (17)	0.24364 (15)	0.0470 (4)	
H23	−0.2047	0.6174	0.3006	0.056*	
N1	0.41798 (16)	0.83035 (15)	0.02676 (13)	0.0575 (4)	
N3	0.32235 (12)	0.48890 (12)	0.12161 (10)	0.0369 (3)	
N2	0.07765 (13)	0.47154 (12)	0.34909 (11)	0.0403 (3)	
H2A	0.0321	0.4158	0.4043	0.048*	
O1	0.18680 (13)	1.05308 (10)	0.26729 (11)	0.0532 (3)	
O2	0.11877 (11)	0.66834 (10)	0.47960 (9)	0.0429 (2)	
O3	0.53230 (11)	0.71535 (13)	0.35228 (10)	0.0496 (3)	
H3A	0.5622	0.6968	0.4269	0.074*	
O4	0.31198 (11)	0.35561 (11)	0.41684 (10)	0.0493 (3)	

### Atomic displacement parameters (Å^2^)

**Table:**

	*U*^11^	*U*^22^	*U*^33^	*U*^12^	*U*^13^	*U*^23^
C1	0.0380 (8)	0.0409 (7)	0.0372 (7)	−0.0030 (6)	0.0020 (6)	−0.0189 (6)
C2	0.0414 (9)	0.0560 (9)	0.0579 (9)	−0.0047 (7)	0.0053 (7)	−0.0260 (8)
C3	0.0431 (10)	0.0754 (12)	0.0775 (12)	0.0055 (9)	−0.0012 (9)	−0.0356 (10)
C4	0.0646 (13)	0.0602 (11)	0.0767 (12)	0.0199 (9)	−0.0136 (10)	−0.0281 (10)
C5	0.0707 (13)	0.0436 (9)	0.0613 (10)	0.0050 (8)	−0.0045 (9)	−0.0160 (8)
C6	0.0520 (9)	0.0413 (8)	0.0410 (7)	−0.0027 (7)	0.0008 (7)	−0.0165 (6)
C7	0.0455 (9)	0.0446 (8)	0.0467 (8)	−0.0126 (7)	0.0116 (7)	−0.0164 (6)
C8	0.0342 (7)	0.0403 (7)	0.0323 (6)	−0.0093 (6)	0.0042 (5)	−0.0154 (5)
C9	0.0376 (8)	0.0378 (7)	0.0325 (6)	−0.0091 (6)	0.0048 (5)	−0.0166 (5)
C10	0.0297 (7)	0.0438 (7)	0.0327 (6)	−0.0089 (6)	0.0032 (5)	−0.0126 (5)
C11	0.0269 (7)	0.0380 (7)	0.0326 (6)	−0.0074 (5)	0.0047 (5)	−0.0115 (5)
C12	0.0348 (8)	0.0444 (7)	0.0386 (7)	−0.0127 (6)	0.0084 (6)	−0.0172 (6)
C13	0.0285 (7)	0.0478 (8)	0.0459 (8)	−0.0025 (6)	0.0049 (6)	−0.0169 (6)
C14	0.0399 (8)	0.0427 (8)	0.0497 (8)	−0.0022 (6)	0.0080 (6)	−0.0186 (6)
C15	0.0573 (11)	0.0598 (10)	0.0600 (10)	−0.0207 (8)	0.0081 (8)	−0.0346 (8)
C16	0.0301 (7)	0.0357 (6)	0.0299 (6)	−0.0070 (5)	0.0047 (5)	−0.0107 (5)
C17	0.0373 (8)	0.0382 (7)	0.0343 (6)	−0.0106 (6)	0.0047 (6)	−0.0121 (5)
C18	0.0316 (7)	0.0435 (7)	0.0381 (7)	−0.0100 (6)	0.0032 (5)	−0.0177 (6)
C19	0.0291 (7)	0.0401 (7)	0.0341 (6)	−0.0080 (5)	0.0017 (5)	−0.0152 (5)
C20	0.0400 (8)	0.0462 (8)	0.0389 (7)	−0.0083 (6)	−0.0015 (6)	−0.0108 (6)
C21	0.0446 (9)	0.0515 (9)	0.0534 (9)	0.0006 (7)	−0.0133 (7)	−0.0145 (7)
C22	0.0314 (8)	0.0645 (10)	0.0673 (10)	−0.0005 (7)	−0.0066 (7)	−0.0325 (9)
C23	0.0298 (8)	0.0627 (10)	0.0569 (9)	−0.0133 (7)	0.0075 (6)	−0.0289 (8)
N1	0.0658 (10)	0.0599 (8)	0.0469 (7)	−0.0260 (7)	0.0109 (7)	−0.0093 (6)
N3	0.0354 (6)	0.0406 (6)	0.0387 (6)	−0.0081 (5)	0.0067 (5)	−0.0183 (5)
N2	0.0363 (7)	0.0448 (6)	0.0405 (6)	−0.0161 (5)	0.0109 (5)	−0.0104 (5)
O1	0.0591 (7)	0.0378 (5)	0.0605 (7)	−0.0115 (5)	0.0125 (5)	−0.0122 (5)
O2	0.0431 (6)	0.0395 (5)	0.0455 (5)	−0.0111 (4)	0.0140 (4)	−0.0120 (4)
O3	0.0338 (6)	0.0765 (7)	0.0456 (6)	−0.0193 (5)	0.0021 (4)	−0.0244 (5)
O4	0.0472 (6)	0.0489 (6)	0.0416 (5)	−0.0078 (5)	−0.0029 (5)	−0.0006 (5)

### Geometric parameters (Å, °)

**Table:**

C1—C6	1.389 (2)	C13—H13B	0.9700
C1—C2	1.397 (2)	C14—N3	1.4663 (18)
C1—C9	1.4682 (19)	C14—H14A	0.9700
C2—C3	1.376 (2)	C14—H14B	0.9700
C2—H2	0.9300	C15—N3	1.4562 (18)
C3—C4	1.388 (3)	C15—H15A	0.9600
C3—H3	0.9300	C15—H15B	0.9600
C4—C5	1.361 (3)	C15—H15C	0.9600
C4—H4	0.9300	C16—N3	1.4750 (16)
C5—C6	1.388 (2)	C16—C19	1.5070 (18)
C5—H5	0.9300	C16—C17	1.5658 (17)
C6—O1	1.3714 (19)	C17—O4	1.2281 (16)
C7—C8	1.3401 (19)	C17—N2	1.3389 (18)
C7—O1	1.3464 (18)	C18—C23	1.375 (2)
C7—H7	0.9300	C18—C19	1.3952 (18)
C8—C9	1.4506 (18)	C18—N2	1.4047 (17)
C8—C10	1.5137 (19)	C19—C20	1.3838 (19)
C9—O2	1.2346 (15)	C20—C21	1.393 (2)
C10—O3	1.4202 (16)	C20—H20	0.9300
C10—C11	1.5545 (17)	C21—C22	1.378 (2)
C10—H10	0.9800	C21—H21	0.9300
C11—C12	1.4806 (18)	C22—C23	1.388 (2)
C11—C13	1.5536 (18)	C22—H22	0.9300
C11—C16	1.5577 (18)	C23—H23	0.9300
C12—N1	1.1370 (18)	N2—H2A	0.8600
C13—C14	1.525 (2)	O3—H3A	0.8200
C13—H13A	0.9700		
			
C6—C1—C2	118.17 (14)	N3—C14—H14A	110.8
C6—C1—C9	119.37 (13)	C13—C14—H14A	110.8
C2—C1—C9	122.46 (13)	N3—C14—H14B	110.8
C3—C2—C1	120.25 (16)	C13—C14—H14B	110.8
C3—C2—H2	119.9	H14A—C14—H14B	108.8
C1—C2—H2	119.9	N3—C15—H15A	109.5
C2—C3—C4	119.87 (18)	N3—C15—H15B	109.5
C2—C3—H3	120.1	H15A—C15—H15B	109.5
C4—C3—H3	120.1	N3—C15—H15C	109.5
C5—C4—C3	121.35 (17)	H15A—C15—H15C	109.5
C5—C4—H4	119.3	H15B—C15—H15C	109.5
C3—C4—H4	119.3	N3—C16—C19	113.02 (10)
C4—C5—C6	118.53 (17)	N3—C16—C11	99.38 (10)
C4—C5—H5	120.7	C19—C16—C11	120.44 (11)
C6—C5—H5	120.7	N3—C16—C17	110.39 (10)
O1—C6—C5	116.38 (14)	C19—C16—C17	101.30 (10)
O1—C6—C1	121.81 (13)	C11—C16—C17	112.55 (10)
C5—C6—C1	121.81 (16)	O4—C17—N2	126.16 (12)
C8—C7—O1	125.13 (14)	O4—C17—C16	125.96 (12)
C8—C7—H7	117.4	N2—C17—C16	107.66 (11)
O1—C7—H7	117.4	C23—C18—C19	122.99 (13)
C7—C8—C9	119.49 (13)	C23—C18—N2	127.48 (13)
C7—C8—C10	120.12 (12)	C19—C18—N2	109.50 (12)
C9—C8—C10	120.38 (11)	C20—C19—C18	118.68 (13)
O2—C9—C8	121.90 (12)	C20—C19—C16	132.64 (12)
O2—C9—C1	123.19 (12)	C18—C19—C16	108.66 (11)
C8—C9—C1	114.91 (12)	C19—C20—C21	118.85 (14)
O3—C10—C8	111.26 (11)	C19—C20—H20	120.6
O3—C10—C11	104.83 (10)	C21—C20—H20	120.6
C8—C10—C11	113.27 (10)	C22—C21—C20	121.21 (15)
O3—C10—H10	109.1	C22—C21—H21	119.4
C8—C10—H10	109.1	C20—C21—H21	119.4
C11—C10—H10	109.1	C21—C22—C23	120.75 (15)
C12—C11—C13	107.68 (11)	C21—C22—H22	119.6
C12—C11—C10	107.99 (10)	C23—C22—H22	119.6
C13—C11—C10	112.06 (10)	C18—C23—C22	117.45 (14)
C12—C11—C16	108.36 (10)	C18—C23—H23	121.3
C13—C11—C16	102.03 (10)	C22—C23—H23	121.3
C10—C11—C16	118.22 (10)	C15—N3—C14	113.60 (12)
N1—C12—C11	177.45 (15)	C15—N3—C16	116.64 (11)
C14—C13—C11	105.22 (11)	C14—N3—C16	106.96 (10)
C14—C13—H13A	110.7	C17—N2—C18	111.97 (11)
C11—C13—H13A	110.7	C17—N2—H2A	124.0
C14—C13—H13B	110.7	C18—N2—H2A	124.0
C11—C13—H13B	110.7	C7—O1—C6	118.17 (11)
H13A—C13—H13B	108.8	C10—O3—H3A	109.5
N3—C14—C13	104.94 (11)		
			
C6—C1—C2—C3	0.3 (2)	C10—C11—C16—C19	71.10 (15)
C9—C1—C2—C3	−179.39 (14)	C12—C11—C16—C17	−171.44 (11)
C1—C2—C3—C4	−0.9 (3)	C13—C11—C16—C17	75.12 (12)
C2—C3—C4—C5	0.7 (3)	C10—C11—C16—C17	−48.25 (15)
C3—C4—C5—C6	0.3 (3)	N3—C16—C17—O4	63.93 (17)
C4—C5—C6—O1	179.54 (15)	C19—C16—C17—O4	−176.09 (13)
C4—C5—C6—C1	−1.0 (2)	C11—C16—C17—O4	−46.11 (18)
C2—C1—C6—O1	−179.85 (13)	N3—C16—C17—N2	−110.91 (12)
C9—C1—C6—O1	−0.2 (2)	C19—C16—C17—N2	9.07 (13)
C2—C1—C6—C5	0.7 (2)	C11—C16—C17—N2	139.05 (11)
C9—C1—C6—C5	−179.63 (13)	C23—C18—C19—C20	−2.9 (2)
O1—C7—C8—C9	−4.4 (2)	N2—C18—C19—C20	178.87 (11)
O1—C7—C8—C10	177.16 (13)	C23—C18—C19—C16	178.50 (12)
C7—C8—C9—O2	−168.83 (13)	N2—C18—C19—C16	0.27 (14)
C10—C8—C9—O2	9.61 (19)	N3—C16—C19—C20	−65.67 (18)
C7—C8—C9—C1	10.87 (18)	C11—C16—C19—C20	51.41 (19)
C10—C8—C9—C1	−170.68 (11)	C17—C16—C19—C20	176.24 (14)
C6—C1—C9—O2	171.04 (13)	N3—C16—C19—C18	112.66 (12)
C2—C1—C9—O2	−9.3 (2)	C11—C16—C19—C18	−130.27 (12)
C6—C1—C9—C8	−8.66 (18)	C17—C16—C19—C18	−5.43 (13)
C2—C1—C9—C8	171.01 (13)	C18—C19—C20—C21	2.6 (2)
C7—C8—C10—O3	29.78 (17)	C16—C19—C20—C21	−179.22 (14)
C9—C8—C10—O3	−148.65 (11)	C19—C20—C21—C22	−0.6 (2)
C7—C8—C10—C11	−88.01 (15)	C20—C21—C22—C23	−1.3 (2)
C9—C8—C10—C11	93.56 (14)	C19—C18—C23—C22	1.0 (2)
O3—C10—C11—C12	−63.12 (13)	N2—C18—C23—C22	178.92 (13)
C8—C10—C11—C12	58.35 (14)	C21—C22—C23—C18	1.1 (2)
O3—C10—C11—C13	55.30 (13)	C13—C14—N3—C15	−161.39 (12)
C8—C10—C11—C13	176.78 (11)	C13—C14—N3—C16	−31.23 (14)
O3—C10—C11—C16	173.51 (11)	C19—C16—N3—C15	−56.75 (15)
C8—C10—C11—C16	−65.02 (15)	C11—C16—N3—C15	174.33 (11)
C13—C11—C12—N1	−49 (3)	C17—C16—N3—C15	55.90 (15)
C10—C11—C12—N1	72 (3)	C19—C16—N3—C14	174.84 (11)
C16—C11—C12—N1	−159 (3)	C11—C16—N3—C14	45.93 (12)
C12—C11—C13—C14	−89.80 (13)	C17—C16—N3—C14	−72.51 (13)
C10—C11—C13—C14	151.59 (11)	O4—C17—N2—C18	175.49 (13)
C16—C11—C13—C14	24.15 (13)	C16—C17—N2—C18	−9.68 (15)
C11—C13—C14—N3	2.87 (14)	C23—C18—N2—C17	−171.89 (13)
C12—C11—C16—N3	71.75 (12)	C19—C18—N2—C17	6.24 (16)
C13—C11—C16—N3	−41.69 (11)	C8—C7—O1—C6	−5.0 (2)
C10—C11—C16—N3	−165.06 (10)	C5—C6—O1—C7	−173.25 (14)
C12—C11—C16—C19	−52.08 (15)	C1—C6—O1—C7	7.3 (2)
C13—C11—C16—C19	−165.52 (11)		

### Hydrogen-bond geometry (Å, °)

**Table:**

*D*—H···*A*	*D*—H	H···*A*	*D*···*A*	*D*—H···*A*
N2—H2A···O2^i^	0.86	2.01	2.8479 (14)	164.
O3—H3A···O4^ii^	0.82	1.97	2.7631 (14)	164.
C23—H23···O3^iii^	0.93	2.58	3.2761 (18)	133.

Symmetry codes: (i) −*x*, −*y*+1, −*z*+1; (ii) −*x*+1, −*y*+1, −*z*+1; (iii) *x*−1, *y*, *z*.

## References

[bb1] Bello, C., Cea, M., Dal Bello, G., Garuti, A., Rocco, I., Cirmena, G., Moran, E., Nahimana, A., Duchosal, M. A., Fruscione, F., Pronzato, P., Grossi, F., Patrone, F., Ballestro, A., Dupuis, M., Sordat, B., Nencioni, A. & Vogel, P. (2010). *Bioorg. Med. Chem.* **18**, 3320–3334. 10.1016/j.bmc.2010.03.009 20346684

[bb2] Bruker (2007). *APEX2* and *SAINT* Bruker AXS Inc., Madison Wisconsin, USA.

[bb3] Cremer, D. & Pople, J. A. (1975). *J. Am. Chem. Soc.* **97**, 1354–1358.

[bb4] Farrugia, L. J. (1997). *J. Appl. Cryst.* **30**, 565.

[bb5] Nardelli, M. (1983). *Acta Cryst.* C**39**, 1141–1142.

[bb6] Pettersson, M., Campbell, B. M., Dounary, A. B., Gray, D. L., Xie, L., O’Donnell, C. J., Stratman, N. C., Zoski, K., Drummond, E., Bora, G., Probert, A. & Whisman, T. (2011). *Bioorg. Med. Chem. Lett.* **21**, 865–868. 10.1016/j.bmcl.2010.11.066 21185183

[bb7] Selvanayagam, S., Sridhar, B., Ravikumar, K., Saravanan, P. & Raghunathan, R. (2011). *Acta Cryst.* E**67**, o629. 10.1107/S1600536811004880 PMC3052033 21522383

[bb8] Sheldrick, G. M. (2008). *Acta Cryst.* A**64**, 112–122. 10.1107/S0108767307043930 18156677

[bb10] Sheldrick, G. M. (1996). *SADABS* University of Göttingen, Germany.

[bb9] Spek, A. L. (2009). *Acta Cryst.* D**65**, 148–155. 10.1107/S090744490804362X PMC2631630 19171970

